# Liraglutide and polycystic ovary syndrome: is it only a matter of body weight?

**DOI:** 10.1007/s40618-023-02084-6

**Published:** 2023-04-24

**Authors:** G. Pugliese, G. de Alteriis, G. Muscogiuri, L. Barrea, L. Verde, F. Zumbolo, A. Colao, S. Savastano

**Affiliations:** 1 Department of Clinical Medicine and Surgery, Endocrinology Unit, University of Naples “Federico II”, Naples, Italy; 2 Department of Humanities, Telematic University Pegaso, Naples, Italy; 3 Unesco Chair “Education for Health and Sustainable Development”, University “Federico II” Naples, Via Sergio Pansini 5, 80131 Naples, Italy

**Keywords:** Polycystic ovary syndrome, Glucagon-like peptide-1 receptor agonists, Liraglutide, Obesity, Weight loss

## Abstract

Despite Polycystic Ovary Syndrome (PCOS) is a very prevalent disorder among women of reproductive age, there is widespread agreement that until now, no pharmacological options are available to tackle the entire spectrum of clinical manifestations encountered in the clinical practice. Obesity and insulin resistance, which commonly characterized this syndrome, prompted the design of studies investigating the effects of glucagon-like peptide 1 (GLP-1) receptor agonists (GLP-1RA) in PCOS. Indeed, a very impressive number of randomized controlled clinical trials (RCTs) and systematic reviews provided robust evidence on the effectiveness of GLP-1RA in PCOS as a new, appealing approach, producing both satisfactory and permanent weight loss, and improvement of insulin resistance at the same time. However, most of the subjects included in the RCTs are PCOS patients with obesity/overweight, whereas a portion of PCOS women, which can even reach 50%, might present a lean phenotype. Moreover, some benefits on clinical and metabolic features of PCOS may not have fully emerged due to the low or medium doses employed in the vast majority of the current studies. Thus, pitfalls in the methodology of these studies have led sometimes to misleading results. In addition, some aspects of GLP-1 beyond weight loss, such as preclinical evidence on GLP-1 effects in directly modulating the hypothalamus–pituitary–gonadal axis, or the effects of GLP-1RA on clinical and biochemical expression of hyperandrogenism, still deserve a greater insight, especially in light of a possible therapeutic use in PCOS women independently of obesity. Aim of this review is to further unravel the possible role of GLP-1 in PCOS pathogenesis, tempting to provide additional supports to the rationale of treatment with GLP-1RA in the management of PCOS also independent of weight loss. For this purpose, the outcomes of RCTs investigating in PCOS the anthropometric and metabolic changes have been treated separately to better underpin the effects of GLP-1 RA, in particular liraglutide, beyond weight loss.

## Introduction

PCOS is the most common endocrinopathy affecting women of reproductive age, with a prevalence of 8–13% depending on diagnostic criteria used and population studied [1, 2]. Overall, PCOS is characterized by oligomenorrhea, anovulation, clinical and/or biochemical hyperandrogenism, and polycystic ovarian morphology, variably combined to generate different diagnostic criteria, among which the most used are the Rotterdam criteria [3].

Although it is not part of the diagnostic criteria, obesity is a common finding in women with PCOS, with a prevalence that ranges between 50 and 80% [4]. Obesity contributes to the reproductive and metabolic abnormalities characterizing this syndrome. In this regard, a link between PCOS and metabolic syndrome has been documented in several studies, showing a prevalence of metabolic syndrome among women with PCOS ranging from 33 to 46% [5–7].

The main hormonal alterations found in PCOS include increased luteinizing hormone/follicle stimulating hormone (LH/FSH) ratio, hyperandrogenism, and insulin resistance, with consequent compensatory hyperinsulinemia, occasionally independent of body weight [2, 8]. Hyperinsulinemia is closely involved in key steps of the pathogenesis of PCOS and hyperandrogenism [9–12]. In particular, increased plasma insulin concentrations stimulate the gonadotropin releasing hormone (GnRH) pulsatility and potentiate the LH-dependent effect on the ovarian theca cells, leading to the enhanced synthesis of androgens and to the arrest of pre-antral follicle development [13]. In addition, hyperinsulinemia is responsible for the increase in the androgen production through both the direct stimulation of insulin receptors and the cross-reaction with Insulin-like Growth Factor-1 (IGF-1) receptors expressed on thecal cells [14]. Moreover, increased plasma insulin levels also increase the amount of circulating free androgens by reducing the production of Sex Hormone-Binding Globulin (SHBG) in the liver [15]. The insulin resistance in PCOS might also occur independently of body weight, due to inherited post-receptor defects in insulin signalling that affects metabolism and mitotic pathways, not only in classic insulin target tissues, such as skeletal muscle, liver, and adipose tissue, but also in the non-classical target tissues, such as pituitary and ovary [12, 16]. In this complex scenario, the concomitant presence of obesity further increases insulin resistance and exacerbates the clinical presentation of PCOS. In particular, the aromatization of circulating androgens to estrone in adipose tissue depots amplifies the deregulation of gonadotropin secretion with worsening of hyperandrogenism. In addition, hyperandrogenism by itself stimulates the deposition of adipose tissue in the visceral area, with further increase in insulin resistance, thus contributing to the vicious circle [17]. Weight loss that is preferably achieved with a multicomponent lifestyle intervention including diet, regular exercise and behavioural strategies, represents one of the main therapeutic tools of PCOS management. Weight loss and lifestyle changes have proven to be effective in restoring menstrual regularity and reproductive function, improving hyperandrogenism, glucose and lipid metabolism, and insulin sensitivity, thus reducing cardiovascular risk in women with PCOS [18–20]. However, only in a minority of PCOS patients with overweight/obesity, a 5–10% weight loss goal is achieved with lifestyle intervention alone. In 2018, the recommendations of the international evidence-based guidelines for the assessment and management of PCOS [1] indicated that, although its use is generally off label, metformin can be considered in addition to lifestyle in women with PCOS and Body Mass Index (BMI) ≥ 25 kg/m^2^ to potentiate weight loss and to improve endocrine and metabolic outcomes. Indeed, in clinical practice, metformin demonstrated a variety of beneficial effects on menstrual disorder, anovulation, androgen excess, insulin resistance, and cardiovascular risk, whereas its weight loss effect is often inconsistent and transient [21, 22].

The issue of obesity and insulin resistance prompted the design of a number of studies investigating the effects of antidiabetic drugs in PCOS, as reported in recent and very exhaustive reviews on the available clinical trials on glucagon-like peptide 1 receptor agonists (GLP-1RA) in PCOS [21, 23, 24].

Considering the network that bi-directionally links obesity and insulin resistance in the pathogenesis of PCOS, it seems to be particularly appealing a new approach producing, at the same time, satisfactory and permanent weight loss, improvement in insulin resistance and in metabolic profile, with additional favourable outcomes on reproductive and cardiovascular systems (19). Nevertheless, there is widespread agreement that until now no pharmacological options are available to tackle the entire spectrum of clinical manifestations observed in PCOS [21]. In addition, preclinical evidence on GLP-1 effects in directly modulating the hypothalamus–pituitary–gonadal (HPG) axis, or the effects of GLP-1RA on clinical and biochemical expression of hyperandrogenism, concern new aspects in PCOS which are probably not always related to weight loss. These effects still deserve a greater insight, especially in the light of a possible therapeutic use in PCOS women independently of obesity. Contrariwise, other relevant aspects, such as the safety profile and tolerability of GLP-1RA or the beneficial effects on fertility in PCOS, have been more extensively treated, especially considering that possible direct or indirect reproductive effects of GLP-1 RA might require a concomitant effective contraception and a washout period before pregnancy [22, 24–31].

Aim of this review is to give experimental and clinical supports on the possible role of GLP-1 in PCOS pathogenesis and treatment, independently on its weight-loss effect. For this purpose, we firstly analyzed the effects of GLP-1 on HPG axis, particularly considering its actions at both central (hypothalamus and pituitary) and peripheral (ovary) levels. Afterwards, we highlighted the limited clinical evidence provided by the studies investigated the GLP-1 secretion in normal-weight women with PCOS. Finally, to better underpin the GLP-1 RA effects, we reported some of the complementary and alternative effects of GLP-1 RA, in particular liraglutide, which go beyond its impact on weight loss in PCOS. In details, we evidenced separately anthropometric parameters, metabolic effects, changes in biochemical and clinical hyperandrogenism, body composition, and ectopic fat that have been reported in the available randomized controlled clinical trials (RCTs) in PCOS women with overweight/obesity.

## Glucagon-like peptide-1 and HPG axis

GLP-1 is an intestinal hormone secreted postprandially from the intestinal L cells in the distal ileum and proximal colon in response to nutrients intake. GLP-1 exerts its anorectic effect in part by acting on the hunger-satiety centre located in the arcuate nucleus of the hypothalamus, and in part by delaying gastric emptying. GLP-1 plays important physiological roles in regulating the glucose homeostasis stimulating the pancreatic secretion of insulin and inhibiting glucagon secretion [32]. However, the wide distribution of GLP-1 receptor (GLP-1R) in numerous cells prompted the development of the studies investigating possible effects of GLP1 on different organs [33].

The anatomical distribution of GLP-1R throughout the reproductive system and several preclinical evidence support that GLP-1 can modulate the HPG axis activity at different levels, thus connecting reproductive functions, metabolic system, nutrition, and energy homeostasis mechanisms.

There is growing evidence that GLP-1 and GLP-1 RA might play a strategic role in mammalian reproduction as a new metabolic signal in the reproductive system; moreover, their effects on the HPG axis could go beyond a mere weight reduction [33].

### HPG axis central effects: hypothalamus and pituitary

Evidence on the potential neuroendocrine actions of GLP-1 on HPG axis was provided by an in vitro study in a rodent hypothalamic neuronal cell line (GT1-7) that reported a dose-dependent stimulating effect of GLP-1 on GnRH release, in association with a prompt increase in the plasma LH levels following the intracerebroventricular injection of GLP-1 in male rats [34].

In an in vivo study, the acute treatment with GLP-1 in female rats resulted in the increase of the amplitude of the preovulatory LH surge, likely by the activation of the kisspeptin hypothalamic system, a key regulator of the pulsatile release of GnRH from GnRH neurons [35]. In addition, the estradiol and progesterone secretion were stimulated, and the number of Graafian follicles and corpora lutea were increased along the estrous cycle [35]. Conversely, the chronic exposure to the GLP-1RA Exendin-4 (Ex4) reduced the hypothalamic expression of the kisspetptin-1 (Kiss-1) receptors, with a marked reduction of the preovulatory LH peak and a delayed puberty onset [35].

An in vitro study using GnRH neurons of adult male mice demonstrated that both GLP-1 and Ex4 might excite GnRH neurons, either directly or indirectly by modulating the nitric oxide (NO) and endocannabinoid pathways that control the presynaptic excitatory GABA-ergic inputs to GnRH neurons [36].

An experimental model of GLP-1R knockout mice (GLP-1R-/-) [37] demonstrated that (GLP-1R-/-) mice exhibited only modest decreases in gonadal weight, while females exhibited delayed puberty, but subsequent fertility and sex-steroid secretion were normal. These results prompted the hypothesis on a direct role of GLP-1 in controlling pubertal development through the hypothalamic GnRH secretion, although this mechanism still remains to be unraveled.

More recently, Arbabi et al.[38] demonstrated the effect of both GLP-1 and Ex4 in an experimental model of steroid-treated ovariectomized ewes. The direct administration of GLP-1 and Ex4 into the median eminence, at the level of the secretory terminals of GnRH neurons, stimulated the GnRH secretion with concomitant stimulation of LH secretion, at least in the ovine species.

The pituitary gland is located outside the blood–brain barrier and expresses GLP-1 R in both rats and humans [39]. However, a significant direct effect of GLP-1 on pituitary LH release is less likely. In fact, the expression of GLP-1R mRNA in the pituitary was lower than in the hypothalamus and even declined during the estrous cycle [35].

While the inhibitory effects exerted by Ex4 on the HPG axis in some species might seemingly contradict its potential use of in humans, the role of more potent and longer lasting GLP-1 RA deserves further investigations.

### HPG axis peripheral effects: ovary

Starting from the observation that GLP-1 promoted proliferation and survival in pancreatic β-cell, He W et al. detected the GLP-1R expression in both human ovarian cancer tissues and ovarian cell lines by immunohistochemical analysis [40]. Of interest, Ex4 was effective in both inhibiting proliferation, migration, and invasion of ovarian cancer cells and enhancing their apoptosis through the inhibition of the PI3K/Akt pathway. It is well established that the apoptosis-induced follicular atresia of the granulosa cells (GCs) is also a key component of the failure in the dominant follicle selection [41]. The role of GLP-1 in mediating the function of GCs and the oocyte development in PCOS was investigated by Sun Z et al. in an experimental mouse model of PCOS [42]. Mice were randomly divided into two groups (vehicle and PCOS-induced groups). In this part of the study, the authors demonstrated the GLP-1R was expressed in mural granulosa cells (MGCs), and its expression was decreased in the PCOS model. A subgroup of the PCOS-induced mice received liraglutide 0.2 mg/kg for 21 consecutive days (twice daily, s.c.), while the other subgroup was treated with saline injections twice daily. The administration of liraglutide significantly attenuated PCOS-associated ovarian MGCs apoptosis in a concentration-dependent manner, and these effects were associated to modifications of the phosphorylation sites of forkhead box protein O1 (FoxO1), a negative regulator of cell survival. Compared to the placebo group, in the liraglutide group the body weight reduced significantly, and up to 40% mice recovered regular estrous cycles, but testosterone levels were only slightly decreased and the reduction in fasting insulin was not statistically significant. These observations lend support to the hypothesis that GLP-1/GLP-1R axis might directly act on ovarian MGCs to promote the follicular development in a dose-dependent manner, likely contributing to the oocyte maturation in PCOS.

More recently, Nishiyama Y et al. investigated the effects of incretins, including GIP (gastric inhibitory polypeptide) and GLP-1, on ovarian steroidogenesis on rat primary GCs. The Authors reported that treatment with incretins significantly suppressed the FSH-induced synthesis of progesterone through the inhibition of the expression of progestogenic factors and enzymes, whereas there was no significant effect on oestrogen synthesis by rat granulosa cells, thus suggesting the possible role of GLP-1 RA in the treatment of dysregulated steroidogenesis in PCOS [43].

In addition, Artunc-Ulkumen B et al. previously reported that glucose toxicity affected severely ovary and endometrium in streptozocin-induced diabetic rats [44]. In particular, diabetes-related hyperglycemia acts through oxidative stress, fibrosis and severe inflammation in the reproductive tract, as demonstrated by the increase in serum transforming growth factor beta (TGF-ß), malonylaldehyde (MDA), and pentraxin-3 (PTX-3), with reduction in ovarian reserve and antimullerian hormone (AMH). Of interest, in the group of streptozocin-induced diabetic rats, the treatment with Ex4 reduced inflammation and fibrosis both in the ovary and endometrium, with associated decrease in inflammatory and oxidative stress markers, and increased ovarian reserve.

**Fig. 1:**
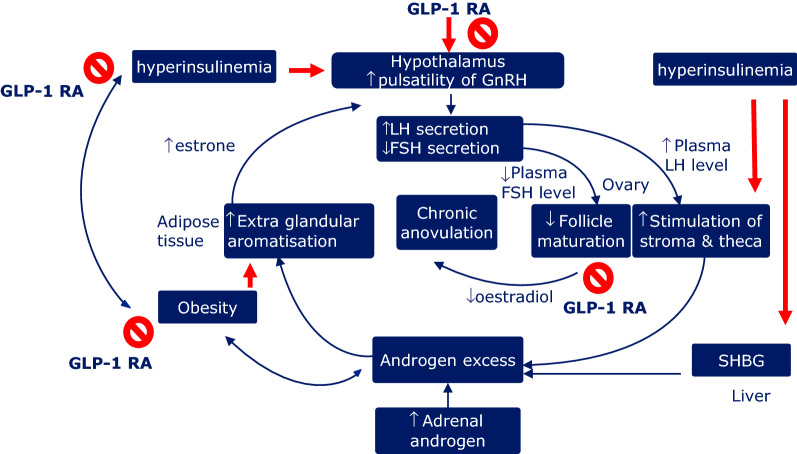
Disruption of the HPG axis in PCOS and possible beneficial effects of GLP-1 RA. *GLP-1 RA,* glucagon-like peptide-1 receptor agonists; *GnRH*, gonadotropin releasing hormone; *LH*, luteinizing hormone; *FSH*, follicle stimulating hormone; *SHBG*, Sex hormone-binding globulin

The disruption of the HPG axis in PCOS and the possible beneficial effects of GLP-1 RA are depicted in Fig. 1.

Legend to Fig. 1. In addition to the well-known effects of GLP-1 and GLP-1 RA in improving insulin sensitivity and reducing body weight, at central levels, GLP-1 and GLP-1 RA might exert regulating effects on GhRH release, and on concomitant gonadotropin secretion, likely by activating the kisspeptin hypothalamic system, a key regulator of the pulsatile release of GnRH from GnRH neurons. At peripheral levels, the administration of GLP-1 RA regulates follicular proliferation and apoptosis-induced follicular atresia of the granulosa cells, a key component of the dominant follicle selection, possibly contributing to the oocyte maturation in PCOS.

## GLP-1 in normal-weight PCOS

Two studies investigated the GLP-1 secretion in normal-weight women with PCOS compared with age- and BMI-matched healthy women [45, 46]. In the first study, the Authors observed that GLP-1 levels were similar in PCOS and controls in the early phase of the 75-g oral glucose tolerance test (OGTT) until 60 min, but that GLP-1 levels were significantly lower after 180 min, suggesting that women with PCOS might have reduced GLP-1 secretion in the late postprandial phase [45]. In the second study, the Authors investigated fasting and post-meal levels of GLP-1 and found that both were significantly reduced in women with PCOS compared to controls. This finding pinpointed an altered dynamic of incretin secretion in PCOS that might contribute to the risk of type 2 diabetes [46]. Although it has not yet been reported, it is reasonable to expect that this defective GLP-1 secretion could be even more marked in women with PCOS and obesity.

### RCTs of liraglutide in PCOS

Liraglutide is a long-acting human GLP-1 RA currently approved for weight loss management in patients with obesity [47]. Liraglutide is also positioned as a first-line anti-obesity agent for the effective prevention of obesity-related cardiovascular diseases in people with obesity [48].

Liraglutide is widely used in the treatment of type 2 diabetes mellitus at the maximum dose of 1.8 mg/day. The weight loss observed in diabetic subjects led to investigate the potential use of this molecule as anti-obesity drug. The effectiveness of once-daily subcutaneous liraglutide administration for weight loss management in adults was established in five large-scale randomized multicentre phase 3 trials [49–53], four of which were part of the SCALE program (Satiety and Clinical Adiposity—Liraglutide Evidence in nondiabetic and diabetic individuals), leading to its approval at a dose of 3.0 mg subcutaneous once-daily (weekly increased from 0.6 mg to 3 mg/day). Liraglutide is indicated as an adjunct to a reduced-energy diet and increased physical activity for weight loss management in adults with a BMI ≥ 30 kg/m^2^ or BMI of ≥ 27 kg/m^2^ and at least one weight-related comorbidity, including type 2 diabetes mellitus, hypertension, dyslipidaemia, or obstructive sleep apnoea.

Several RCTs evaluated the efficacy of liraglutide *vs.* placebo, metformin, or combinations of liraglutide and metformin at different doses in premenopausal PCOS women with overweight or obesity. The outcomes of these studies mainly concern weight loss and the improvement of anthropometric parameters. Metabolic and endocrine changes, including restoration of menstrual cyclicity or improvement of hyperandrogenism, have also been investigated as secondary outcomes. Table 1 summarizes the RCTs that measured the weight-reducing effects and the other secondary outcomes of GLP-1RA in PCOS patients.

**Table 1:** Main characteristics of the included randomized controlled clinical trials

Author	Country	PCOS Criteria	Participant characteristics	Age (years)	BMI (kg/m^2^)	Study arms	Duration (weeks)	Outcomes					
								Weight changes	Metabolic changes	endocrine changes	Menstrual pattern	US	Body composition
Frøssing et al. 2018 [54]	Denmark	RC	Women with overweight and/or insulin resistance	29.9 ± 6.1	33.3 ± 4.9	(1) LIRA 1.8 mg (n = 48) (2) Placebo (n = 24)	26	Mean weight, BMI, WC and W/H decreased in the LIRA group compared with the placebo group	LIRA caused reduction in fasting glucose, HbA1c and leptin, whereas HOMA2-IR, Matsuda index, insulin, adiponectin and glucagon levels remained unchanged	In the LIRA group, SHBG increased and free testosterone decreased compared with placebo	NE	NE	LIRA caused reduction in total fat and lean body mass (DXA), VAT and SAT (MRI), reduced liver fat content by 44%
Frøssing et al. 2018 [55]	Denmark	RC	Women with overweight and/or insulin resistance	29.9 ± 6.1	33.3 ± 4.9	(1) LIRA 1.8 mg (n = 48) (2) Placebo (n = 24)	26	Mean weight and BMI decreased in the LIRA group compared with the placebo group	HbA1c decreased in the LIRA group compared with the placebo group	NE	NE	NE	NE
Nylander et al 2017 [56]	Denmark	RC	Women with overweight and/or insulin resistance	29.9 ± 6.1	33.3 ± 4.9	(1) LIRA 1.8 mg (n = 48) (2) Placebo (n = 24)	26	Mean weight, BMI and WC decreased in LIRA group compared with the placebo group	NE	NE	NE	NE	NE
Jensterle et al. 2015 [57]	Slovenia	RC	Premenopausal women with obesity	30.7 ± 7.9	38.6 ± 6.0	(1)MET: 1000 mg BID (n = 13) (2)LIRA 1.2 mg s.c (n = 14)	12	LIRA was superior to MET in reducing weight, BMI, and WC	HOMA-IR decreased in both treatment arms LIRA was superior to MET in reducing glucose levels at 120 min during OGTT	No differences in biochemical hyperandrogenism Clinical hyperandrogenism not evaluated	Menstrual frequency increased with both treatments	NE	LIRA resulted in significant decrease in VAT area (DXA)
Jensterle et al. 2015 [58]	Slovenia	NICHD criteria	Premenopausal women with obesity with newly diagnosed PCOS	27.6 ± 7.2	39.5 ± 6.2	(1)MET: 1000 mg BID (n = 14) (2)LIRA 1.2 mg s.c (n = 14)	12	Weight, BMI, WC decreased with both treatments	No significant Metabolic changes were observed in both groups	Total testosterone and LH decreased in MET, LH increased in LIRA No significant change in FG score	No significant changes in menstrual pattern	NE	Whole-body fat mass decreased with both treatments
Salamun et al. 2018 [59]	Slovenia	RC	Infertile women with obesity and PCOS, at first or second IVF attempt	31.1 ± 4.7	36.7 ± 3.5	(1) MET: 1000 mg BID (n = 14) (2) COMBI: MET 1000 mg BID + LIRA 1.2 mg s.c.(n = 14)	12	Weight, BMI, WC decreased in both groups with no difference between arms	HOMA-IR score, Fasting and post OGTT glucose were significantly reduced only in the COMBI group	SHBG increased in MET and COMBI arms compared to baseline Clinical hyperandrogenism not evaluated	NE	NE	VAT area was reduced in all treatment groups without differences between the groups
Jensterle et al. 2015 [60]	Slovenia	RC	Premenopausal women with obesity	37.2 ± 4.5	30.3 ± 4.4	(1) COMBI: MET 1000 mg BID + LIRA 1.2 mg s.c. (n = 22) (2) LIRA 1.2 mg s.c. (n = 21)	12	Weight, BMI and WC reductions were observed in both groups COMBI was superior to LIRA in reducing weight and BMI	Only in COMBI there were reductions in glucose (0 and 120 min during OGTT).COMBI was more effective than LIRA in reducing glucose levels 120 min during OGTT. LIRA therapy was associated with a reduction in basal insulin and a trend of reduction in HOMA-IR	Free testosterone reduced and SHBG increased in LIRA and COMBI groups	NE	NE	VAT mass, VAT volume and VAT area reductions compared with pre-treatment values were observed in both groups
Jensterle et al. 2014 [61]	Slovenia	NICHD criteria	Nondiabetic women with obesity who had lost ≥ 5% of body weight during pre-treatment with MET for at least 6 months	31.3 ± 7.1	37.1 ± 4.6	(1) MET: 1000 mg BID (n = 14) (2) LIRA: 1.2 mg s.c. (n = 11) (3) COMBI: MET 1000 mg BID + LIRA 1.2 mg s.c.(n = 11)	12	COMBI was superior to LIRA and MET monotherapy in reducing weight, BMI, and WC	COMBI was superior to MET in reducing glucose levels at 120 min during OGTT	COMBI induced a significant decrease in androstenedione level Clinical hyperandrogenism not evaluated	No significant changes in menstrual pattern	NE	All treatment interventions resulted in a not significant reduction in VAT area (DXA)
Elkind-Hirsch et al. 2022 [62]	Louisiana	NIH Criteria	Premenopausal women with obesity	1)LIRA 3.0 mg: 41.6 ± 0.9 2)Placebo:43.9 ± 1.5	1)LIRA 3.0 mg: 31 ± 0.8 2)Placebo: 32 ± 1.1	(1) LIRA 3.0 mg (n = 55) (2) Placebo (n = 27)	32	Mean weight, BMI, WC and W/H decreased in the LIRA 3 mg group compared with the placebo group	TRG and TRG/HDL ratio,fasting blood glucose, mean blood glucose during OGTT, HOMA-IR, SIOGTT,IGI/HOMA were improved in LIRA 3 mg group *vs.* placebo	LIRA 3 mg resulted in a significantly decreased FAI compared with placebo	Menses occurrence was significantly increased in LIRA 3 mg group vs placebo	NE	Total fat mass percentage and android/ginoid ratio (DXA) decreased with LIRA 3 mg *vs.* placebo
Jensterle et al. 2017 [63]	Slovenia	RC	Premenopausal women with obesity	33.1 ± 6.1	38.3 ± 5.4	(1) COMBI: MET 1000 mg BID + LIRA 1.2 mg s.c. (n = 15) (2) LIRA 3 mg s.c (n = 15)	12	BMI and WC reduction in LIRA 3 mg was greater than in COMBI	In COMBI reduction of glucose at 0 and 120 min, insulin at 0 min, HOMA-IR and LDL cholesterol In LIRA 3 mg improvement of glucose and insulin levels at 120 min COMBI was superior in the decrease of LDL cholesterol	Only COMBI significantly reduced total testosterone Significant increase of SHBG was observed in only LIRA 3 mg There were no significant between treatment differences in endocrine parameters	NE	NE	NE
Nylander et al. 2017 [64]	Denmark	RC	Women with overweight and/or insulin resistance	29.9 ± 6.1	33.3 ± 4.9	(1) LIRA 1.8 mg (n = 48) (2) Placebo (n = 24)	26	Mean weight decreased in the LIRA group compared with placebo group	Fasting glucose and HbA1c decreased in the LIRA group compared with the placebo group	In the LIRA group SHBG increased, and free testosterone and FAI decreased compared with placebo. No effect on FG score in either group	Bleeding ratio significantly improved with LIRA vs placebo	Ovarian volume decreased in LIRA group	NE

*PCOS* polycystic ovary syndrome, *BMI* body mass index, *US* ultrasound, MET metformin, *BID* twice a day, *LIRA* Liraglutide, *WC*: Waist Circumference, *HoMA-IR* Homeostatic Model Assessment for Insulin Resistance, *VAT* Visceral Adiposity Tissue, *DXA* dual-energy X-ray absorptiometry, *NICHD* National Institute of Child Health and Human Development, *LH* Luteinizing hormone, *FG* Ferriman Gallwey, *LDL* Low-Density Lipoprotein, *SHBG* Sex Hormone binding globulin, *SAT* Subcutaneous adipose tissue, *MRI* Magnetic resonance imaging, *SIOGTT* OGTT-derived insulin sensitivity index, *IGI/HOMA* corrected early insulin response to a glucose challenge, *TRG* triglycerides, *HDL* High-Density Lipoprotein, *IVF* in vitro Fertilization, *RC* Rotterdam Criteria, *NE* Not evaluated

### Anthropometric changes

Some studies compared the liraglutide 1.8 mg/day *vs.* placebo for 26 weeks and demonstrated a greater efficacy in terms of reduction of body weight and waist circumference (WC) in the liraglutide group [54–56]. In particular, in a RCT in women with PCOS with BMI ≥ 25 kg/m^2^ and/or insulin resistance, Frøssing et al. showed that 26 weeks of liraglutide 1.8 mg/day treatment *vs.* placebo not only reduced body weight by 5.2 kg (5.6%), but also visceral adipose tissue (VAT) by 18% [54]. Comparing the efficacy of liraglutide 1.2 mg/day *vs* metformin 1000 mg BID (twice daily), Jensterle et al. carried out two 12-week studies offering different results: the first study showed that liraglutide was superior to metformin in reducing body weight, BMI, and WC [57], while in the second study, both the treatments proved to be equally effective with no differences between the groups [58]. However, in the subgroup of patients with insulin resistance, severe obesity and higher odds ratio for the metabolic syndrome, BMI decreased more with liraglutide than with metformin (2.13 kg/m^2^ vs. 0.62 kg/m^2^, respectively), suggesting a greater effect of liraglutide in subjects with insulin resistance and higher metabolic risk profile [58].

The combined therapy with liraglutide and metformin (COMBI) seems to be more effective. Salamun et al. investigated infertile PCOS patients with obesity randomly assigned to metformin 1000 mg BID or to metformin 1000 mg BID combined with low-dose liraglutide 1.2 mg/day for 12 week, and demonstrated that weight, BMI, and WC decreased with both treatments, without significant difference between the two arms [59]. Instead, Jensterle et al. demonstrated that metformin as an initial adjunct to low-dose liraglutide enhanced the weight-loss potential of liraglutide in PCOS patients with obesity. In fact, the addition of metformin 1000 mg BID to liraglutide 1.2 mg/day was more effective than liraglutide alone in reducing body weight and BMI after 12 weeks of treatment [60]. Interestingly, the same Authors enrolled nondiabetic women with obesity and PCOS who had lost < 5% body weight during pre-treatment with metformin and randomized them into three treatment arms: metformin 1000 mg BID (MET: *n* = 14); liraglutide: 1.2 mg/day (LIRA: *n* = 11); COMBI: metformin 1000 mg BID + liraglutide 1.2 mg/day (COMBI: *n* = 11) [61]. They observed that COMBI therapy was superior to LIRA and MET monotherapy in reducing weight, BMI, and WC in women with PCOS previously poor responders to weight loss on metformin monotherapy. These results supported the hypothesis that an early multi-targeting approach in PCOS may be more effective and may allow the use of low-dose regimens, thus improving both the safety and the tolerability of the treatment and reducing costs. Currently, only two RCTs have evaluated the efficacy of liraglutide at the approved dosage of 3.0 mg/day for the therapy of obesity. A recent randomized, placebo-controlled, phase 3 study conducted by Elkind-Hirsch et al. showed that liraglutide 3.0 mg/day significantly decreased body weight, BMI, WC and Waist to Hip ratio (W/H) compared with the placebo group [62]. Furthermore, Jensterle et al.in another study demonstrated that liraglutide 3.0 mg/day was more effective in reducing BMI and WC in PCOS patients with obesity than the combination of metformin 1000 mg BID + liraglutide at low doses (1.2 mg/day), that has hitherto been considered one of the more promising treatment [63]. Therefore, it might be useful to expand the evidence on high-dose liraglutide, also considering that there are no direct studies that compare high-dose liraglutide and metformin.

### Metabolic changes

In a double-blind, placebo-controlled RCT conducted by Frøssing et al. [54] in women with PCOS, BMI ≥ 25 kg/m^2^ and/or insulin resistance, treatment with liraglutide 1.8 mg/day for 26 weeks, along with a higher weight loss, induced significant reductions in fasting plasma glucose, HbA1c, and plasma glucose AUC, whereas Homeostatic Model Assessment for Insulin Resistance (HOMA-IR), Matsuda index and insulin AUC remained unchanged. In line with this study, in a double-blind, placebo-controlled RCT enrolling PCOS women with BMI ≥ 25 kg/m^2^, Nylander et al. showed that liraglutide 1.8 mg/day for 6 months, along with a mean weight loss of 5.2 kg, was effective in significantly reduce fasting glucose and HbA1c, while HOMA-IR and other insulin resistance parameters remained unchanged [64]. Conversely, the randomized, placebo-controlled, phase 3 study by Elkind-Hirsch et al. demonstrated, in addition to higher weight loss in the group treated with high-dose liraglutide 3.0 mg/day for 32 weeks, significant improvements not only of fasting glucose and HOMA-IR, but also of the post-load glucose measured by OGTT, OGTT-derived insulin sensitivity index (SIOGTT), and corrected the early insulin response to a glucose challenge (IGI/HOMA) compared to placebo [62]. The two studies by Jensterle et al. comparing the short-term monotherapy with liraglutide 1.2 mg/day *vs.* metformin 1000 mg BID in PCOS women with obesity that have been previously reported, also evaluated the efficacy on glucose metabolism [57, 58]. In particular, the first study demonstrated that, along with improvements in anthropometric parameters, HOMA-IR reduced in both treatment arms, but liraglutide 1.2 mg/day was superior to metformin also in reducing glucose levels at 120 min during OGTT [57]. However, the same Authors in the second study, failed to demonstrate a reduction in HOMA-IR, fasting glucose and insulin and glucose and insulin during OGTT in both treated groups [58]. Of interest, in another above-mentioned study by Jensterle et al. comparing liraglutide 1.2 mg/day + metformin 1000 mg BID combination therapy and liraglutide 1.2 mg/day alone in nondiabetic women with PCOS and obesity, again despite a significant improvement in anthropometric parameters in both groups, the combined therapy was associated with a significant reduction in fasting and 120 min glucose levels during OGTT [60]. In the study by Jensterle et al. comparing metformin, liraglutide, and combination therapy (metformin 1000 mg BID + liraglutide 1.2 mg/day), the latter, and not liraglutide 1.2 mg/day alone, was superior to metformin alone in reducing glucose levels at 120 min during OGTT [61]. Of interest, in the randomized trial by Jensterle et al. comparing liraglutide 1.2 mg/day + metformin 1000 mg BID with liraglutide 3 mg/day, the combination therapy was effective in reducing fasting glucose and insulin, HOMA-IR, and LDL cholesterol, while only the high dose of liraglutide resulted in a significant improvement of 120 min during OGTT glucose and insulin levels along with a higher weight loss [63].

### Biochemical and clinical hyperandrogenism

The effects of liraglutide alone or in combination with metformin focusing on biochemical and clinical hyperandrogenism in PCOS were scarcely reported in only few clinical studies among those previously reported, which provided also discordant results.

Frøssing et al. [54] and, more recently, Nylander et al. [64] in their above-mentioned studies demonstrated the effects on hyperandrogenism of liraglutide alone at 1.8 mg/day *vs.* placebo. In particular, in both studies, liraglutide 1.8 mg/day induced a significant increase in SHBG and a decrease in free testosterone. In the second study, the Authors investigated also changes in the clinical expression of hyperandrogenism, but they failed to find any effects on the Ferriman Gallwey (FG) score, probably due the short duration of the study (26 weeks) [64]. The study by Elkind-Hirsch et al. using liraglutide 3 mg/day compared with placebo, despite the lack of changes in total testosterone levels, confirmed the increase in SHBG levels, with a consequent reduction in free Androgen Index (FAI), and also a restoration in menstrual cyclicity [62]. These findings lend support to the hypothesis that the increase in SHBG levels induced by liraglutide might contribute per se to reduce the risk of developing the metabolic syndrome in PCOS, also considering the effect of SHBG in directly suppressing inflammation and lipid accumulation in macrophages and adipocytes [65].

The two studies by Jensterle et al. comparing the efficacy of liraglutide 1.2 mg/day *vs.* metformin 1000 mg BID led to conflicting results on hyperandrogenism. In the first study, the authors did not find any significant difference in biochemical hyperandrogenism in both intra and inter-groups, despite the significant improvement in anthropometric parameters in the group treated with liraglutide [57]. In the second study, the authors reported a significant reduction of total testosterone and LH in only metformin group, while treatment with liraglutide led to a significant LH increase, without differences in FG score between the two groups [58]. However, a number of pitfalls in the methodology of these studies (e.g. small sample size, blood sampling on non-specific day of the menstrual cycle, very short duration of the treatment), might give rise to misleading results.

Focusing on the effects of combination therapy on hyperandrogenism, Jensterle et al. demonstrated that although the combination therapy (metformin 1000 mg BID + liraglutide 1.2 mg/day) resulted in a greater weight loss than liraglutide alone, both groups showed an increase in SHBG and a decrease in free testosterone, with no differences between the two treatment arms, thus suggesting that the effect on the biochemical hyperandrogenism of liraglutide may be partially independent of the extent of weight loss [60]. However, when comparing the combined therapy (metformin 1000 mg BID + liraglutide 1.2 mg/day) with metformin alone, Salamun et al. observed that weight loss and significant increases in SHBG occurred in both groups [59]. Contrariwise, in the study by Jensterle et al. evaluating metformin, liraglutide, and combination therapy (metformin 1000 mg BID + liraglutide 1.2 mg/day), only the latter treatment resulted in significant decrease in body weight and androstenedione levels [61]. It is conceivable that the design of these studies, mainly the low dose of liraglutide used and the short duration follow-up, failed to clearly separate the direct effect of liraglutide on hyperandrogenism from the weight loss derivative one. Indeed, by comparing the high-dose liraglutide therapy (3.0 mg/day) with the combination therapy (metformin 1000 mg BID + liraglutide 1.2 mg/day), Jensterle et al. demonstrated that the high-dose liraglutide therapy was effective in increasing SHBG levels, while low-dose combination therapy resulted also in a decrease in total testosterone despite the minor effect on BMI [63]. Lastly, the results of a very recent systematic review and network meta-analysis aimed to compare the effects of oral antidiabetic drugs in endocrine and metabolic profiles in patients with PCOS confirmed that metformin was more beneficial in reducing serum total testosterone, whereas GLP-1RA + metformin reduced FAI more effectively [24].

These findings could suggest that the effect of liraglutide on hyperandrogenism does not depend exclusively on weight loss, it can also occur at low doses, and that there is probably a synergistic action with metformin.

### Body composition and ectopic fat

VAT accumulation is strictly linked to various metabolic disturbances and obesity-related cardiovascular diseases [66]. Thus, targeting anti-obesity medications to reduce VAT are of strategical importance to tackle efficaciously obesity-related consequences.

Some evidence showed that liraglutide treatment in PCOS resulted in improvements in body composition, with reduction of VAT. In particular, Frøssing et al. demonstrated that a 5.6% weight loss in the group treated with liraglutide 1.8 mg/day compared with placebo was associated to reductions in both total fat and lean body mass measured by Dual-Energy X-ray Absorptiometry (DXA), as well as in visceral and subcutaneous adipose tissues measured by Magnetic Resonance Imaging (MRI) [54]. Furthermore, compared with placebo, liraglutide treatment decreased leptin by 20% and liver fat content by 44%, thus supporting a specific effect on hepatic ectopic fat. These Authors failed to evidence significant changes in indexes of insulin resistance and speculated that other classical effects of GLP-1 RA, such as suppressed glucagon secretion, with consequent reduced hepatic glucose production, could have been involved in the improvement in glucose metabolism and ectopic fat deposition [54].

These effects on body composition were recently confirmed by Elkind-Hirsch et al. at the maximal dose of 3.0 mg/day [62]. In this study, measurements of DXA revealed that total fat mass percentage and android/ginoid ratio decreased with liraglutide 3.0 mg/day compared with placebo.

The studies of comparison between low-dose liraglutide and metformin 1000 mg BID have yielded scant and contradictory evidence on the effective superiority of liraglutide in reducing VAT. On one side, a study showed that body weight and whole-body fat mass decreased with both treatments, but without difference between the two arms [58]. On the other side, another study of the same Authors showed that liraglutide 1.2 mg/day determined a significant decrease in VAT area measured by DXA, and at the same time a greater reduction in body weight and BMI compared to metformin 1000 mg BID [57]. The studies evaluating the efficacy of combined low-dose liraglutide with metformin *vs.* metformin alone or *vs.* liraglutide at low doses alone, did not demonstrate the greater efficacy of the combination therapy, as the area of VAT was reduced in all the three treatments arms without inter-groups differences [59, 60]. The only available study by Jensterle et al. comparing liraglutide 3.0 mg *vs.* low-dose liraglutide combination therapy with metformin did not include the body composition assessment [63]. However, although currently there are no comparing studies on the efficacy of different doses of liraglutide in improving body composition, it is tempting to speculate that greater changes in body composition might be expected through administration of higher doses of liraglutide.

## Conclusions

The available results have not yet provided a precise therapeutic indication. GLP-1 RA, mainly liraglutide, might represent interesting molecules to tackle the weight excess that is associated with metabolic abnormalities, hyperandrogenism, infertility, and menstrual alterations in PCOS women. However, the question remained on whether the possible beneficial therapeutic actions of GLP-1 RA in PCOS might depend exclusively on the improvement in body weight and metabolic profile or on direct effects on HPG axis in PCOS. Most of the subjects included in the available RCTs are PCOS patients with severe obesity, which do not reflect the entire clinical spectrum of PCOS encountered in clinical practice. In addition, in the vast majority of these studies, liraglutide was used at low or medium doses, while it is well known that some effects, in particular weight loss, are dose-dependent and more pronounced at the maximum dose. Therefore, some benefits on clinical and metabolic features of PCOS may not have fully emerged from the current studies, suggesting the need for additional RCTs, to expand the evidence on high-dose liraglutide effects. Finally, the potential effectiveness of new generation GLP-1 RA in terms of the dose- and exposure time-response still deserves further explorations.

## Data Availability

Not applicable.
